# iTRAQ-based quantitative proteomic and physiological analysis of the response to N deficiency and the compensation effect in rice

**DOI:** 10.1186/s12864-019-6031-4

**Published:** 2019-08-28

**Authors:** Qiangqiang Xiong, Lei Zhong, Tianhua Shen, Chaohao Cao, Haohua He, Xiaorong Chen

**Affiliations:** 1 Key Laboratory of Crop Physiology, Ecology and Genetic Breeding, Ministry of Education, College of Agronomy, Jiangxi Agricultural University, Nanchang, 330045 China; 2 College of Agronomy, Jiangxi Agricultural University, Nanchang, China; 3 Southern Regional Collaborative Innovation Center for Grain and Oil Crops in China, Changsha, China

**Keywords:** Rice, Yield, iTRAQ, Quantitative proteomic, Compensatory effect

## Abstract

**Background:**

The crop growth compensation effect is a naturally biological phenomenon, and nitrogen (N) is essential for crop growth and development, especially for yield formation. Little is known about the molecular mechanism of N deficiency and N compensation in rice. Thus, the N-sensitive stage of rice was selected to study N deficiency at the tillering stage and N compensation at the young panicle differentiation stage. In this study, a proteome analysis was performed to analyze leaf differentially expressed proteins (DEPs), and to investigate the leaf physiological characteristics and yield under N deficiency and after N compensation.

**Results:**

The yield per plant presented an equivalent compensatory effect. The net photosynthetic rate, optimal/maximal quantum yield of photosystem II (Fv/Fm), soil and plant analyzer development (SPAD) value, and glutamic pyruvic transaminase (GPT) activity of T1 (N deficiency at the tillering stage, and N compensation at the young panicle differentiation stage) were lower than those of CK (N at different stages of growth by constant distribution) under N deficiency. However, after N compensation, the net photosynthetic rate, Fv/Fm, SPAD value and GPT activity were increased. Using an iTRAQ-based quantitative approach, a total of 1665 credible proteins were identified in the three 4-plex iTRAQ experiments. Bioinformatics analysis indicated that DEPs were enriched in photosynthesis, photosynthesis-antenna proteins, carbon metabolism and carbon fixation in the photosynthetic organism pathways. Moreover, the photosynthesis-responsive proteins of chlorophyll a-b binding protein, ribulose bisphosphate carboxylase small chain and phosphoglycerate kinase were significantly downregulated under N deficiency. After N compensation, chlorophyll a-b binding protein, NADH dehydrogenase subunit 5, NADH dehydrogenase subunit 7, and peroxidase proteins were significantly upregulated in rice leaves.

**Conclusion:**

Through physiological and quantitative proteomic analysis, we concluded that a variety of metabolic pathway changes was induced by N deficiency and N compensation. GO and KEGG enrichment analysis revealed that DEPs were significantly associated with photosynthesis pathway-, energy metabolism pathway- and stress resistance-related proteins. The DEPs play an important role in the regulation of N deficiency and the compensation effect in rice.

**Electronic supplementary material:**

The online version of this article (10.1186/s12864-019-6031-4) contains supplementary material, which is available to authorized users.

## Background

Elemental nitrogen (N) is one of the main elements of amino acids, chlorophyll, nucleic acids, lipids and many intermediate metabolites. It plays an important role in plant growth and agricultural production [1, 2]. The yield of most crops has increased twice as much as that in the past 30 years, which is not only due to the breeding of new varieties but also the cultivation of innovative management methods including N fertilizer application methods [3, 4]. However, the overuse of N fertilizer in rice production in China and the improper proportion of N fertilizer used in different growth stages lead to a low utilization rate of N fertilizer [5, 6], and less than 50% of N fertilizer is absorbed by plants [7]. The irrational use of N fertilizer not only increases the cost of production but also has negative effects on soil microorganisms, plants, animals and the environment itself [8]. The N metabolic pathway of rice high-yield has not been thoroughly elucidated so far [9]. Especially in cases of improper N management during cultivation due to weather or seasonal drought, it may lead to missed N applications at certain developmental stages. Seasonal drought often occurs in rice regions in southern China, which is often accompanied by the inability of N fertilizer to be applied to the soil [10, 11]. The N fertilizer can not be applied until the water is restored. The yield formation mechanism of N deficiency and the compensation effect in rice is worthy of in-depth research. This subject has important significance for balancing ecological environment protection, high yield and stable yield of crops.

The proteomics technology has been made considerable progress and has been applied to analyze biological problems including N metabolism [12–14]. Protein is the embodiment and executor of plant function, which not only regulates plant stress tolerance by changing the catalytic activity of enzymes, but also regulate the expression of other genes partly as a transcription factor. As macromolecular substances, proteins can regulate the composition and concentration of intracellular substances and then affect the osmotic pressure of plants. In the past ten or twenty years, two mainstream methods of labeling quantification (iTRAQ/tandem mass tag and stable isotope labeling strategies) and label-free methods have been developed. There is also the rapid development of data-independent acquisition technology in recent years. However, the data-independent acquisition technique is suitable for protein detection in large sample sizes and complex systems. Proteomic techniques provide a crucial and complementary tool to dissect the molecular mechanisms underlying crop adaptation to mineral nutrient deficiency in the rapidly progressing postgenome era [15–17]. Proteomics is a powerful approach to gain insight into plant metabolic adaptation to different stimuli. There are two main mechanisms for N metabolism in plants. One mechanism is a high affinity transport system, which is a functional gene that promotes N uptake by promoting constitutive and inducible expression of plants under low-N conditions. The other mechanism is a low affinity transport system, which slows N uptake by plants under high N conditions. These mechanisms have led to significant differences in N uptake and utilization of various crop varieties. Related studies have been reported in such crops as rice (*Oryza sativa* L.), *Zea mays*, *Brassica campestris* and *Triticum aestivum* [14, 18–21]. Previous studies on N deficiency in plants were carried out using proteomic techniques, which indicated that the metabolism of photosynthesis-related proteins, carbohydrates, amino acids and other metabolic pathways changed significantly [22, 23]. However, the limitation of these studies was the rapid response mechanism of plants under N deficiency without involving a compensation mechanism for double N application in the next growth stage after N deficiency.

The crop growth compensation effect is a naturally biological phenomenon. Researchers have noted the physiological and ecological mechanisms of crop water and nutrient deficiency compensation effects [24, 25]. Crop growth compensation effect is a type of adaptive mechanism formed in the long-term environmental change process and implies the ability to promote crop growth and yield formation at morphological and physiological levels [26, 27]. The molecular mechanism underlying N deficiency and N compensation in double-cropping super hybrid rice has not been fully understood, and no report on this topic has been published until now.

Hence, this study used a double-cropping super hybrid early rice (Wufengyou 286) variety as material, and soil with low available N content was selected. iTRAQ-labeled quantitative proteomics was used to explore the molecular mechanism of N deficiency and N compensation effect in rice, especially the remedy for improper N management, and to provide a scientific basis for the high and stable yield of double-cropping early rice. This study also provides a new ecological perspective for the study of N utilization in rice.

## Methods

### Plant materials and growth conditions

Wufengyou 286 (*Oryza sativa* L*.*) is a dominant double-cropping super hybrid early rice (Wufeng A/Zhonghui 286, a super rice variety, certified by China’s Ministry of Agriculture in 2015). Experiments were performed at the Science and Technology Park of Jiangxi Agricultural University in Nanchang, Jiangxi Province of China in 2017 (28°46′N, 115°50′E, altitude: 48.8 m, annual average temperature: 17.5 °C, average annual sunshine: 1720.8 h, annual average evaporation: 1139 mm, and average annual rainfall: 1747 mm). Rice was planted in plastic buckets (height, 24.0 cm; inner diameter of the upper portion, 29.0 cm; inner diameter at the bottom, 23.5 cm). Soil was sampled from the upper soil layer (0–20 cm) of the rice experiment field at the Science and Technology Park of Jiangxi Agricultural University. The physical and chemical properties of the experimental soil were as follows: soil pH 5.76, organic matter content 10.2 mg kg^− 1^, total N 1.8 g kg^− 1^, alkali-hydrolyzable N 36.2 mg kg^− 1^, available phosphorus 4.8 mg kg^− 1^, and available potassium 10.2 mg kg^− 1^. The soil was naturally air-dried, pulverized by soil disintegrator (FT-1000A, Changzhou WIK Instrument Manufacturing Co. Ltd. China) and sieved through a 100-mm mesh. Each pot contained approximately 10 kg dry soil, which was soaked in water two weeks before transplantation. At the four-leaf stage, the rice seedlings that grew consistently well were transplanted into the pots with three seedlings per pot and one seedling per hole. Considering the nutrient demand of rice, 5.0 g calcium-magnesium-phosphate and 1.0 g potassium-chloride were applied in each pot at the tillering and the heading stages. Urea was applied as the N source (3.0 g per pot is equivalent to 120 kg hm^− 2^). According to the experimental design, the fertilizer at seedling stage was mixed in the barrel before transplantation, and N fertilizer for other growth stages was applied by irrigation. The paddy was under unified management before transplantation. After transplantation, the management of water and insects was performed in accordance with a high-yield cultivation mode. The pots were moved under shelter before heavy rain to prevent loss of fertilizer nutrients with overflowing water and were moved back to the net house immediately after the rain. All of the agronomic measurement were followed the advice of local management to avoid losses in production.

### N treatment and sampling time

Based on previous research on N deficiency and its effective compensation threshold in double-cropping super hybrid rice, N deficiency sensitive stage is the tillering stage and N compensation effective stage is the young panicle differentiation stage [25]. CK treatment was 0.6 g N fertilizer applied at each stage (seedling, tillering, young panicle differentiation, heading, and milk maturation stages). T1 treatment was no N fertilizer applied at the tillering stage (0 g) and 1.2 g N fertilizer applied at the young panicle differentiation stage. N fertilizer applied of T1 at the other growth stages (seedling, heading, and milk maturation stages) was 0.6 g. The specific N application schemes are shown in Table 1. According to a randomized block design, each treatment consisted of three replicates, and 20 pots constituted each replication. The time schedule of field management including sowing on 16 March 2017 and transplanting on 27 April. The seedling, tillering, young panicle differentiation, heading, and milk maturation stages occurred on 26 April, 8 May, 7 June, 20 June, and 27 June, respectively. Fresh sample leaves of rice plants with similar growth were obtained before young panicle differentiation stage N application (N deficiency) and 14 days after N compensation at the young panicle differentiation stage. Based on sampling time, the fresh leaves of the two treatments (CK and T1) were divided into four groups. The four groups pertaining to the two treatments were as follows: normal N supply at the tillering stage was sampled from CK at the tillering stage (6 June), N deficiency at the tillering stage was sampled from T1 at the tillering stage (6 June), normal N supply at the panicle differentiation stage was sampled from CK at the young differentiation panicle stage (19 June), and N compensation at the young panicle differentiation stage was sampled from T1 at the young panicle differentiation stage (19 June). Three biological replicates were taken from each group of samples, frozen in liquid N, and stored in a refrigerator at − 80 °C until proteins were extracted.

**Table 1:** Experimental design for N supply

Treatment	Total urea supply	Young seedling stage	Tillering stage	Young panicle differentiation stage	Heading stage	Milk-maturation
CK	3	0.6	0.6	0.6	0.6	0.6
T1	3	0.6	0	1.2	0.6	0.6

CK: N at different stages of growth by constant distribution, T1: N deficiency at the tillering stage, and N compensation at the young panicle differentiation stage

### Net photosynthetic rate

The net photosynthetic rate of rice leaves was measured after N deficiency (June 5) and N compensation (June 22). For each treatment, six plants in a state of good and consistent growth were selected, labeled, and measured for net photosynthetic rate in leaf from the top on the main stem on a sunny day between 9:00–11:00 am using a CI-340 handheld photosynthesis system (CID Bio-Science, USA).

### Chlorophyll fluorescence parameter

The chlorophyll fluorescence parameters of rice leaves were measured by a PM2500 basic modulation chlorophyll fluorescence instrument (CID Bio-Science, USA) after N deficiency (June 5) and N compensation (June 18), and three biological replicates were employed per treatment. After 30 min of dark adaptation, the initial fluorescence F_0_ of the photosystem II reaction center was measured when the reaction center was in an open state, and the stable fluorescence of the photosystem II reaction center was in the closed state under the light adaptation condition. The final calculation shows the optimal/maximal quantum yield of photosystem II (Fv/Fm) [28, 29].

### SPAD value (chlorophyll content)

The Soil and Plant Analyzer Development (SPAD) value of rice leaves was measured under N deficiency (June 6) and N compensation (June 18). For each treatment, six plants in a state of good and consistent growth were selected, labeled, and measured for SPAD in leaf from the top on the main stem. The SPAD value was measured from the base, middle, and top of each leaf using a SPAD-502 chlorophyll analyzer (Zhejiang Tuopu Instrument Co. Ltd., China), and the average SPAD value was calculated.

### Determination of glutamic pyruvic transaminase activity

After N deficiency at the tillering stage (June 5) and N compensation at the young panicle differentiation stage (June 19), the leaf sample of rice was taken 0.1 g for each treatment, and three biological replicates were performed per treatment. All samples were flash-frozen in liquid N and stored at − 80 °C until the activity of glutamic pyruvic transaminase (GPT) in rice leaves was determined [30].

### Yield and yield components

After maturation, five undamaged plants per treatment were selected and harvested to evaluate yield. The yield per plant, effective panicle number per plant, number of total grains per panicle, number of filled grains per panicle, panicle length, and 1000-grain weight were measured, and the seed setting rate was calculated accordingly.

Calculation method of 1000-grain weight: One thousand full-filled seeds were selected randomly for each replicate and weighed separately to obtain the average value;

Seed setting rate = (Number of filled grains per panicle) / (Number of total grains per panicle).

### Statistical analysis

SPSS 17.0 and Origin 8.5 were used to analyze data for significant differences and plotting.

Yield was the primary indicator to evaluate the effects of compensation [31]. Since the crops were planted in pots in this study, the compensation effect was evaluated by yield per plant following a previously described method by which the compensation index was the ratio of yield per plant after N deficiency and N compensation to that of a control plant [32]. The compensation effect was determined in combination with variance test results: compensation index > 1 with a significant variance test result (*p* < 0.05) indicated overcompensation; compensation index equal to or close to 1 with an insignificant variance test result (*p* < 0.05) indicated equivalent compensation; and compensation index < 1 with a significant variance test result (*p* < 0.05) indicated inadequate compensation.

### Chemicals and reagents

Urea, DL-dithiothreitol, iodacetamide, IPG buffer, and formic acid were purchased from GE (USA); SDS (sodium dodecyl sμlfonate), Tris (Tris(hydroxymethyl)aminomethane), TCA (Trichloroacetic acid), AP (Ammonium persμLfate) and tetramethylethylenediamine were purchased from Amresco (USA); TEAB (triethylammonium bicarbonate) and Coomassie Brilliant Blue G-250 were purchased from Sigma (USA); trypsin was purchased from Promega (USA); acetonitrile, HPLC grade and H_2_O were purchased from Thermo (USA).

### Protein extraction, digestion and iTRAQ labeling

**Fig. 1:**
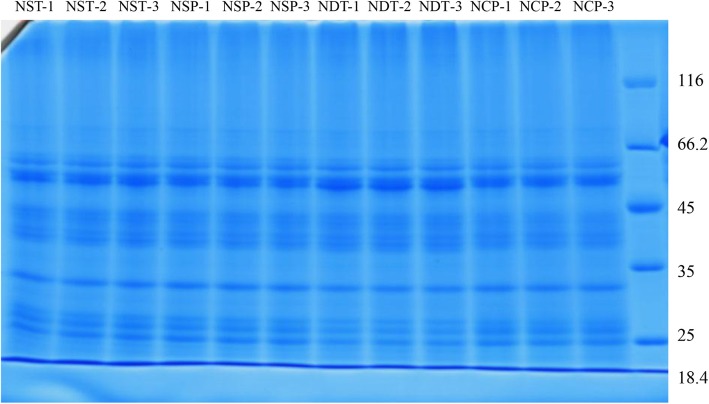
Detection of total protein in leaves of different groups samples at different stages by SDS-PAGE electrophoresis. NST: normal N supply at the tillering stage, NDT: N deficiency at the tillering stage, NSP: normal N supply at the young panicle differentiation stage, NCP: N compensation at the young panicle differentiation stage. NST-1, NST-1 and NST-1; NDT-1, NDT-2 and NDT-3; NSP-1, NSP-2 and NSP-3; NCP-1, NCP-2 and NCP-3 are three replicates between different groups

Protein extraction and digestion were performed using the FASP method, as described by Wisniewski et al. (2009) [33]. After the protein was extracted, 15 μg samples were placed onto a 12% SDS-PAGE gel. The gel was visualized using CBB stain according to Candiano’s protocol [34]. The stained gel was scanned using an image scanner (GE Healthcare, USA). The electrophoresis results showed that the extracted rice leaves had clear protein bands, no degradation and high quality, which could be used in the next stage of the iTRAQ experiment. The SDS-PAGE results are shown in Fig. 1. Then, the resulting peptide mixture was labeled with three 4-plex iTRAQ reagents according to the manufacturer’s instructions (AB Sciex Inc., Foster City, CA, USA). Briefly, 100 μg protein extract was mixed with 120 μL reducing buffer (10 mM DL-dithiothreitol, 8 M urea, 100 mM TEAB, pH 8.0), and the solution was incubated at 60 °C for 1 h. Iodacetamide was added to reach a final concentration of 50 mM in the dark at room temperature for 40 min. The filter units were centrifuged at 12000 rpm (Rotor type: 1.5/2.2 ml × 24 (16,000 r/min)) for 20 min, and the flow-through was discarded from the collection tube. Then, 100 μL 100 mM TEAB was added, and the samples were centrifuged at 12000 rpm for 20 min. This step was repeated twice. The filter units were transferred to new collection tubes, and 100 μL 100 mM TEAB and 2 μL sequencing-grade trypsin (1 μg/μL) were added to the samples, which were incubated at 37 °C for 12 h. The samples were centrifuged at 12000 rpm for 20 min, and the peptide was collected. Then, 50 μL 100 mM TEAB was added, and the tube was centrifuged again. The collected solution was mixed again, and the solution was lyophilized. The sample was reconstituted in 100 μL 100 mM TEAB, and then a 40 μL sample was transferred to a new tube for labeling. Each required vial of iTRAQ reagent was allowed to reach room temperature. The solution was spun to the bottom of the vial, and 200 μL of isopropanol was added. Each vial was vortexed and then spun. The process was repeated one more time. Then, 100 μL of iTRAQ reagent was added to the sample tube. Each vial was vortexed and then spun. The tubes were incubated at room temperature for 2 h, and 200 μL water was added to quench the labeling reaction. The solution was lyophilized, and the samples were stored at − 80 °C until LC-MS/MS analysis.

### LC-MS/MS analysis

The tryptic peptides were fractionated by high pH reverse-phase HPLC using an Agilent Zorbax Extend-C18 column (2.1 × 150 mm, C18, 5 μm, 120 Å, ChromXP Eksigent). Briefly, peptides were eluted at a flow velocity of 300 μL/min. The gradient elution conditions were as follows: 0–8 min, 98% A; 8–8.01 min, 98–95% A; 8.01–38 min, 95–75% A; 38–50 min, 75–60% A; 50~50.01 min, 60–10% A; 50.01–60 min, 10% A; 60–60.01 min, 10–98% A; and 60.01–65 min, 98% A. Samples were collected for 8–50 min, and the eluate was collected into the centrifuge tube every other minute. After collecting and freeze-drying, the samples were frozen and stored on mass spectrometry.

All analyses were performed using a Q-Exactive mass spectrometer (Thermo, USA) equipped with a Nanospray Flex source (Thermo, USA). Samples were loaded onto a capillary C18 trap column (2 cm × 75 μm) and were separated by a C18 column (15 cm × 75 μm) on an EASY-nLC™ 1200 system (Thermo, USA). The flow rate was 300 nL/min, and the linear gradient was 90 min (from 5 to 85% B over 67 min; mobile phase A = 2% acetonitrile/0.1% formic acid and B = 95% acetonitrile/0.1% formic acid). Full MS scans were acquired in the mass range of 300–1800 m/z with a mass resolution of 70,000, and the AGC target value was set at 1000000. The twelve most intense peaks in MS were fragmented with higher-energy collisional dissociation with a collision energy of 28. MS/MS spectra were obtained with a resolution of 35,000 with an AGC target of 50,000 and a max injection time of 100 ms. The Q-E dynamic exclusion was set for 30.0 s and run under positive mode.

### Database search and protein quantification

The raw files were analyzed by Proteome Discoverer™ 1.3 (Thermo Company, USA), and the database was obtained from the UniProt database (https://www.uniprot.org/proteomes/?query=Oryza+sativa&sort=score). The false positive rate of peptide identification was controlled below 1%. The specific search database parameters are set as shown in Table 2.

**Table 2:** Search parameters by mass spectrometry

Sample Type	Cys. Alkylation	Digestion	Instrument	Database
iTRAQ 4-plex (Peptide labeled)	Iodoacetamide	Trypsin	Q Exactive	*Oryza sativa subsp./ glaberrima.fasta*

The relative quantitative protein analysis of samples according to the ratios of iTRAQ reporter ions derived from all unique peptides that represented each protein was conducted using Proteome Discoverer™ 1.3. The relative peak intensities of the iTRAQ reporter ions derived from each of the MS/MS spectra were employed, and the REF sample was used as a reference in calculating the iTRAQ ratios of the reporter ions. The final ratios derived from the relative protein quantifications were normalized according to the median protein quantification ratio. The protein ratios represented the median of the unique peptides in the protein. Only proteins identified in all three replicates were considered for further analysis.

### Bioinformatics analysis

*P*-values < 0.05 were determined by Benjamini-Hochberg false discovery rate in Perseus, and a ratio fold change (FC) of > 1.20 or < 0.83 in expression between any two groups was considered significant. The GO function entries for all alignment protein sequences were extracted using the mapping function of OmicsBean. GO annotation was used to analyze the identified proteins, and the identified proteins were categorized by biological process, molecular function, and cellular component. The DEPs were further analyzed using the Kyoto Encyclopedia of Genes and Genomes (KEGG) database (http://www.genome.jp/kegg/kaas/). The R language software was used to investigate the hierarchical clustering of the identified DEPs and data visualization. Columns were mean-centered, and the Euclidean distance and average linkage were used for data aggregation.

## Results and discussion

### Yield and physiological response to N deficiency and N compensation

**Fig. 2:**
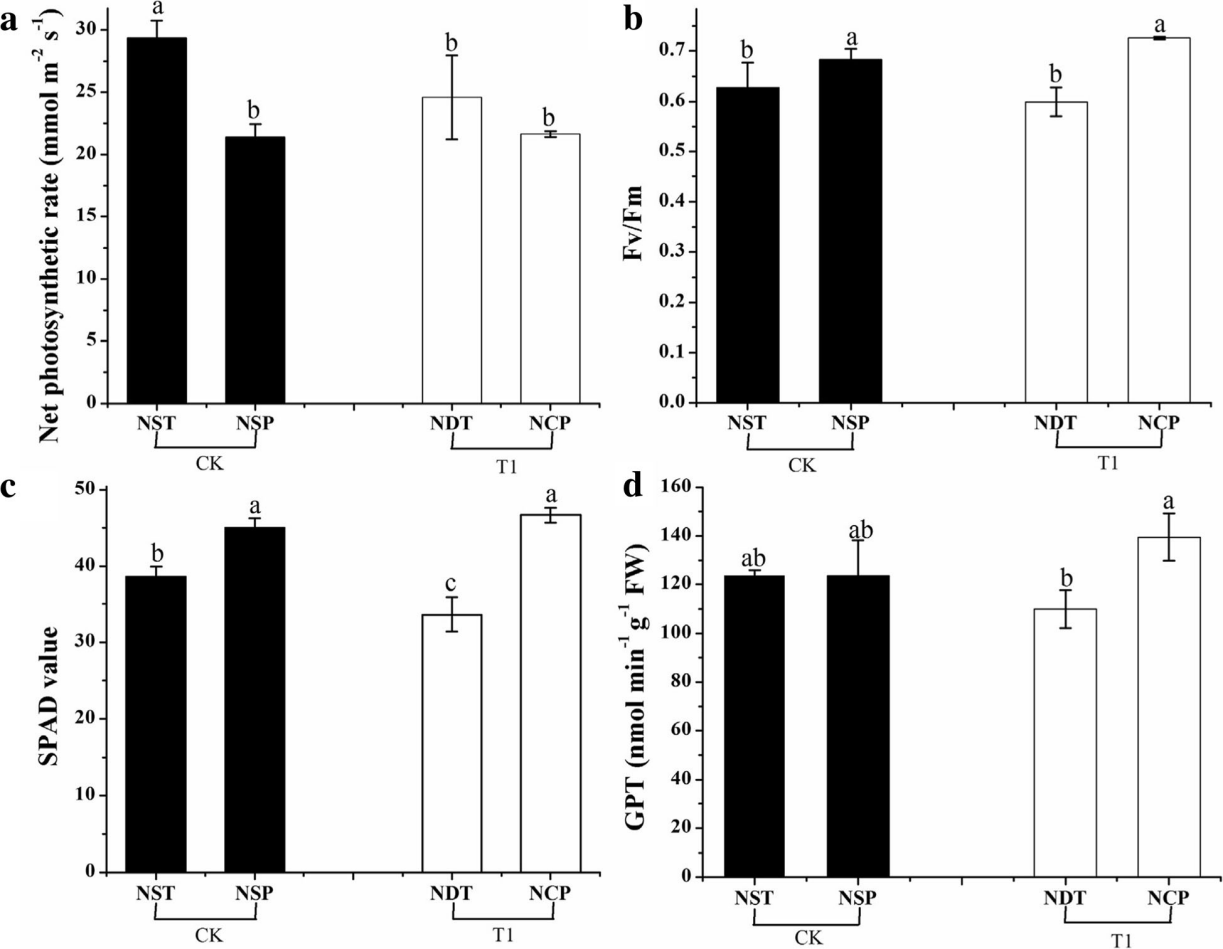
Analysis of net photosynthetic rate, Fv/Fm, SPAD value and GPT activity. (**a**) Net photosynthetic rate, (**b**) Fv/Fm, (**c**) SPAD value, (**d**) GPT activity. The column data in figure is average, and the short lines represent the mean square deviation. Values in the same column show means via Tukey’s multiple range test. Different lowercase letters represent significant differences (*p* < 0.05). NST: normal N supply at the tillering stage, NDT: N deficiency at the tillering stage, NSP: normal N supply at the young panicle differentiation stage, NCP: N compensation at the young panicle differentiation stage

The yield and physiological changes of rice under N deficiency and N compensation were investigated. T1 was 6.48% higher than CK in terms of yield per plant (Table 3), with no significant difference (*p* > 0.05) being observed between T1 and CK. The effective panicle number per plant of T1 was higher than that of CK, and there was a significant difference between them (*p* < 0.05) (Table 3). The number of total grains per panicle was lower than CK, and there was a significant difference between them (*p* < 0.05) (Table 3). The panicle length, seed setting rate and 1000-grain weight of T1 were 0.74, 1.03 and 1.65% lower than those of CK, respectively, with no significant difference between T1 and CK (*p* > 0.05). The compensation index of T1 was 1.07, this was not significantly different to CK. From the compensation index and variance significance, we observed that the compensation effect was equal. The “Tri-control” fertilization technology system of rice established by Zhong et al. (2007) emphasizes the control of the total N application rate and N fertilizer application. The “Tri-control” fertilization technology system emphasizes the proportion of N fertilizer and delaying the application time at the tillering stage should be reduced and the proportion of N fertilizer at the panicle differentiation stage should be increased to ensure high and stable yield of rice [35]. In this study, N deficiency at the tillering stage and N compensation at the young panicle differentiation stage of rice actually supported the core technology of N fertilizer application in this system, moreover it were actually N fertilizer backward shift. The yield was not significantly reduced after the N fertilizer backward shift. The failure to apply sufficient N fertilizer in time due to drought and other reasons at the young panicle differentiation stage can be replenished at the earliest during the panicle differentiation stage, which can ensure stable production. In addition, in regard to topdressing in production practice, it depends on the growth of rice plants and soil fertility. Therefore, the production of double-cropping super hybrid early rice should pay special attention to the application of panicle fertilizer. N plays a vital role in the synthesis of chlorophyll, protein and amino acids in plants; thus, N deficiency seriously affects their synthesis [36, 37]. Chlorophyll fluorescence technology, as a natural probe in crops, can detect considerable crop growth and nutritional status information [38]. The internal light energy conversion efficiency or the maximum light energy conversion efficiency of the photosystem II reaction center can be reflected by the Fv/Fm [39]. The results showed that the net photosynthetic rate (Fig. 2a), Fv/Fm (Fig. 2b) of T1 under N deficiency was lower than that of CK. Compared with NST (normal N supply at the tillering stage), the net photosynthetic rate (Fig. 2a), Fv/Fm (Fig. 2b) of NDT (N deficiency at the tillering stage) decreased by 16.33 and 4.57%, respectively, indicating that N deficiency could somewhat inhibit the growth of rice. After N compensation, the net photosynthetic rate (Fig. 2a) and Fv/Fm (Fig. 2b) of NCP (N compensation at the young panicle differentiation stage) increased by 1.26 and 6.20%, respectively, compared with NSP (normal N supply at the panicle differentiation stage). Compared with NDT, the Fv/Fm (Fig. 2b) of NCP increased significantly (*p* < 0.05) by 21.16%. GPT is a class of transaminases that rely on pyridoxal phosphate as a coenzyme. The available substrates for reversible reactions are pentanedioic acid pyruvate and glutamic acid, which are involved in N assimilation and signal transduction. GPT also plays an indispensable role in carbon and N metabolism in plants. It has been confirmed that GPT can help plants resist stress [40, 41]. Previous studies show that SPAD values of chlorophyll analyzer readings can predict chlorophyll content per unit area of crops such as rice. SPAD values can also be used to estimate the N content per unit weight of leaves, especially N per unit area [42]. The results showed that SPAD value (Fig. 2c) and GPT (Fig. 2d) activity (*p* < 0.05) decreased significantly under N deficiency, SPAD value (Fig. 2c) and GPT (Fig. 2d) activity increased significantly (*p* < 0.05) after N compensation. Compared with NDT, the SPAD value (Fig. 2c) and GPT (Fig. 2d) of NCP increased significantly (*p* < 0.05) by 38.49 and 26.86%, respectively. The changes in these physiological indexes also confirmed the occurrence of a compensation effect. In summary, the rice N deficiency at the tillering stage and N compensation at the young panicle differentiation stage had a certain compensation effect on yield.

**Table 3:** Analysis of yield and yield components

Treatments	Effective panicle number per plant	Panicle length (cm)	Number of total grains per panicle	Seed setting rate^a^ (%)	1000-grain weight (g)	Yield per plant (g)	Compensation index^b^
CK	4.60 ± 0.55	20.16 ± 0.34	129.45 ± 4.09	82.43 ± 0.61	2.42 ± 0.04	17.44 ± 0.46	1.00 ± 0.00
T1	6.40 ± 1.14*	20.01 ± 0.20^ns^	121.98 ± 5.26*	81.58 ± 0.60^ns^	2.38 ± 0.05^ns^	18.57 ± 1.37^ns^	1.07 ± 0.09^ns^

CK: N at different stages of growth by constant distribution, T1: N deficiency at the tillering stage, and N compensation at the young panicle differentiation stage. Values in the same column show mean via Tukey’s multiple range test, ^ns^not significantly different, *Significant difference with *p* < 0.05, *n* = 5

^a^Seed setting rate = (Number of filled grains per panicle) / (Number of total grains per panicle)

^b^CI = (Yield per plant) / (Yield per plant of CK)

### Quantitative proteomic analysis with iTRAQ

The present study conducted an N deficiency and N compensation-induced proteomic experiment by iTRAQ labeling using rice leaves. We performed three 4-plex iTRAQ experiments, analyzing three samples per group at the tillering stage and the young panicle differentiation stage, with each sample corresponding to a pool of three plants. A total of 2308 proteins were identified, and 2091 proteins were quantified in the first 4-plex iTRAQ experiment (Additional file 1: Table S1; Additional file 2: Table S2). In addition, 2428 proteins were identified, and 2201 proteins were quantified in the second 4-plex iTRAQ experiment (Additional file 3: Table S3; Additional file 4: Table S4). Moreover, 2484 proteins were identified, and 2241 proteins were quantified in the third 4-plex iTRAQ experiment (Additional file 5: Table S5; Additional file 6: Table S6). The samples after enzymatic hydrolysis were analyzed by LC-MS, and the database was searched after analysis. Using the criteria of Score Sequest HT > 0 and Unique peptides ≥1 and removing the blank value, the results showed that 2016 credible proteins were screened from iTRAQ the results of experiment 1, 2134 credible proteins were screened from iTRAQ the results of experiment 2, and 2168 credible proteins were screened from iTRAQ the results of experiment 3. A total of 1665 credible proteins were identified in the three 4-plex iTRAQ experiments. These proteins were considered for further analysis.

**Fig. 3:**
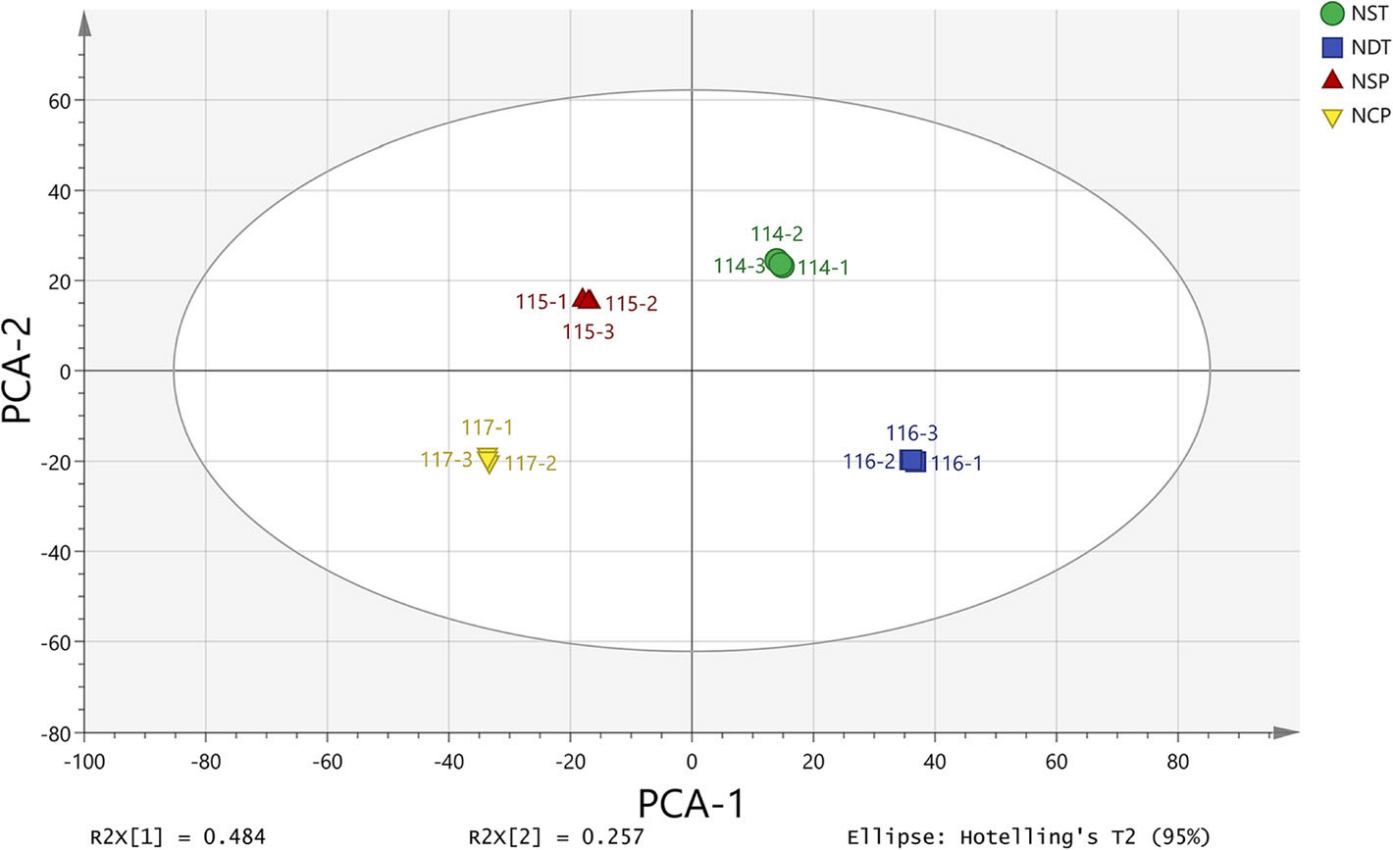
Principal component analysis between different treatment groups using individual replicates. One hundred fourteen is the NST sample, and 116 is the NDT sample. NST: normal N supply at the tillering stage, NDT: N deficiency at the tillering stage. One hundred fifteen is the NSP sample, and 117 is the NCP sample. NSP: normal N supply at the panicle differentiation stage, NCP: N compensation at the young panicle differentiation stage

**Fig. 4:**
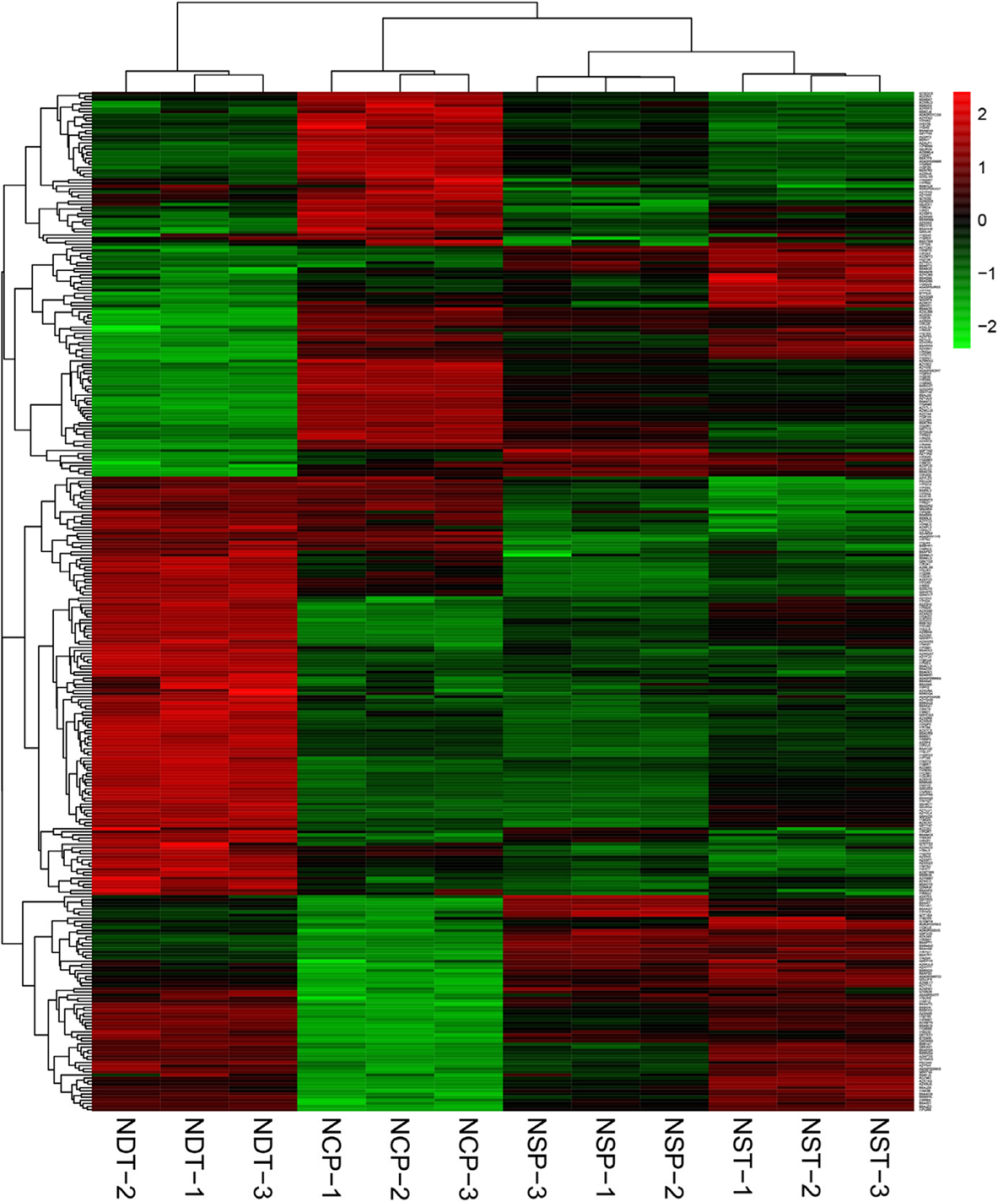
Results of hierarchical cluster analysis of changed proteomics. Hierarchical trees were drawn based on FC in expression. Columns correspond to repetition between NDT, NCP, NSP, and NST, while rows represent DEPs. Red and green colors indicate high and low relative expression of proteins, respectively. NST: normal N supply at the tillering stage, NDT: N deficiency at the tillering stage. NSP: normal N supply at the young panicle differentiation stage, NCP: N compensation at the young panicle differentiation stage

As a starting point for the DEP analysis, expression data were used to determine the global relationship between the different groups. Principle component analysis (PCA) was performed using the individual replicates from each group. The results demonstrated that although the first two principal components could explain 74.1% of the variation, the data points in the graph formed tight clusters between the different groups (Fig. 3). Additionally, we visualized the expression patterns of different points in each group by clustering analysis (Fig. 4), which further indicates that the data are reliable.

**Fig. 5:**
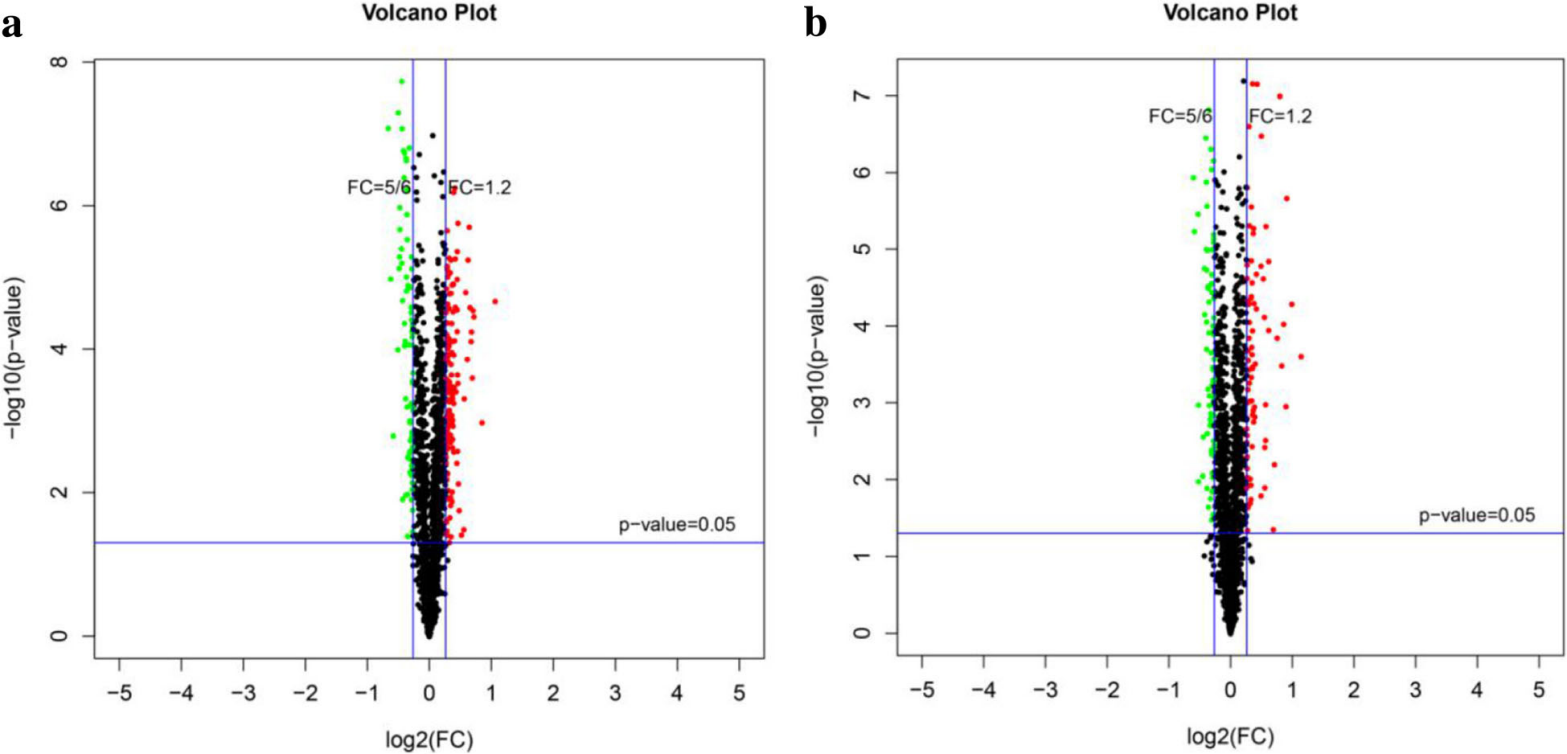
Volcano plot between NDT-NST (**a**), volcano plot between NCP-NSP (**b**). The transverse coordinate of the volcano plot is log_2_(FC), which represents a more significant difference away from the zero point. The longitudinal coordinate is -log(*p*-value), which represents a more significant difference away from the zero point. The green dots indicate downregulated DEPs, the red dots indicate upregulated DEPs, and the black spots indicate nonsignificant DEPs. NST: normal N supply at the tillering stage, NDT: N deficiency at the tillering stage. NSP: normal N supply at the young panicle differentiation stage, NCP: N compensation at the young panicle differentiation stage

**Fig. 6:**
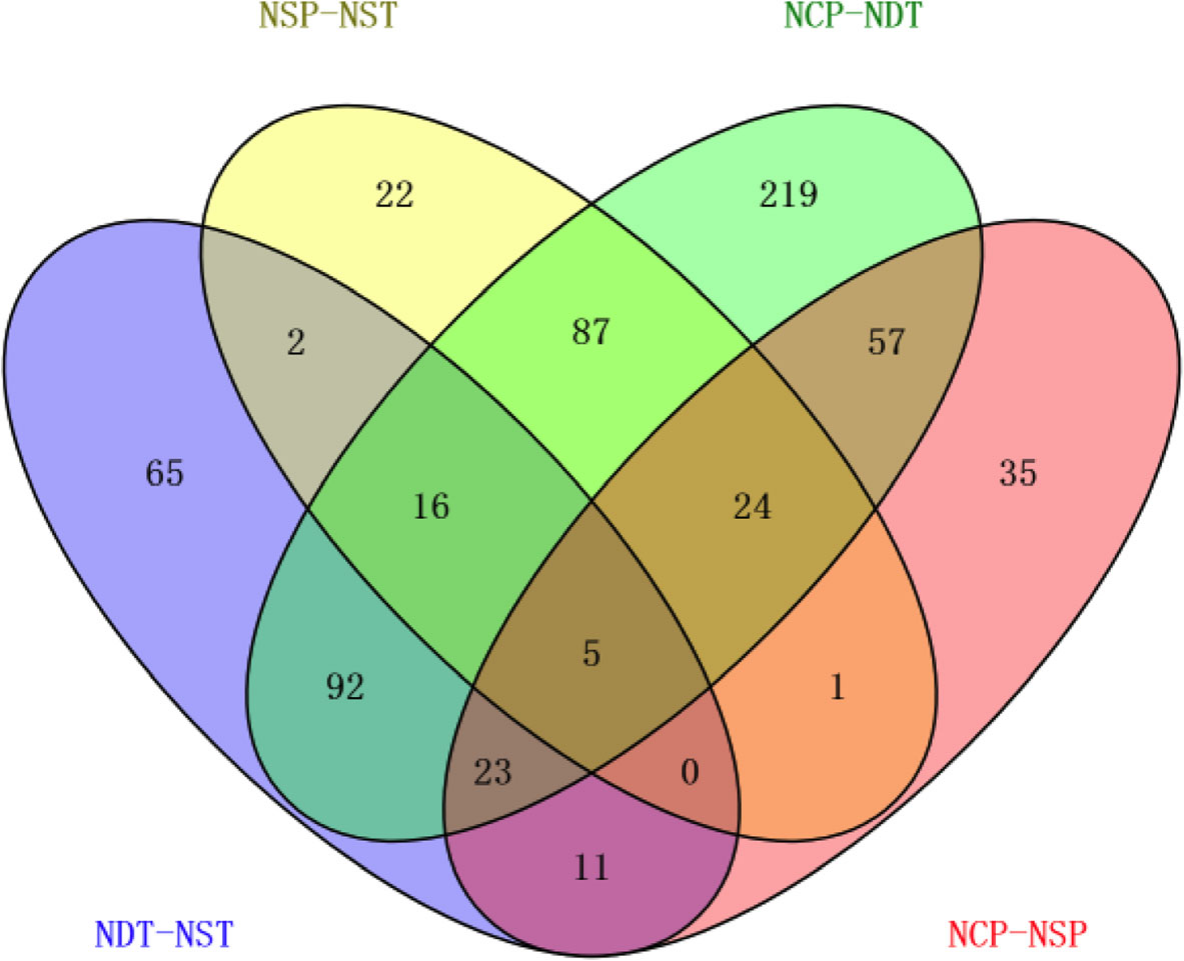
Expression patterns of DEPs in the different comparison groups (NDT-NST, NCP-NSP, NSP-NST, NCP-NDT). NST: normal N supply at the tillering stage, NDT: N deficiency at the tillering stage. NSP: normal N supply at the young panicle differentiation stage, NCP: N compensation at the young panicle differentiation stage

Based on the analysis of selected credible proteins (the results of each group of repeats can be identified by credible proteins), the difference in multiple FC values and the significant *p*-value of each comparison group were calculated. FC > 1.2 or FC < 5/6 and *p*-value < 0. 05 were used as the criteria for screening DEPs. The volcano plot of the DEPs of NDT-NST is shown in Fig. 5a and of NCP-NSP is shown in Fig. 5b. In addition, we could better demonstrate the expression patterns of proteins in the different comparison groups (Fig. 6)

### Functional analysis of DEPs

To further understand how plant respond to N deficiency and N compensation, proteins with FC > 1.2 and *p*-value < 0.05 were regarded as upregulated, whereas those with FC < 5/6 and *p*-value < 0.05 were considered downregulated. The DEPs were hence considered N deficiency- and N compensation-responsive proteins. These cut-offs were selected based on a previous publication that investigated the reproducibility of iTRAQ™ quantification [43]. According to this criterion, there were 214 DEPs between NDT and NST (140 upregulated and 74 downregulated) (Additional file 7: Table S7); 156 DEPs between NCP and NSP (80 upregulated and 76 downregulated) (Additional file 8: Table S8). Of the DEPs between NDT and NST, 55 upregulated proteins and 31 downregulated proteins could be annotated with functions, while 85 upregulated proteins and 43 downregulated proteins remained uncharacterized (Additional file 7: Table S7). Of the DEPs between NCP and NSP, 41 upregulated proteins and 27 downregulated proteins could be annotated with functions, while 39 upregulated proteins and 49 downregulated proteins remained uncharacterized (Additional file 8: Table S8). Of the DEPs between NCP and NDT, 85 upregulated proteins and 117 downregulated proteins could be annotated with functions, while 105 upregulated proteins and 216 downregulated proteins remained uncharacterized (Additional file 9: Table S9).

**Fig. 7:**
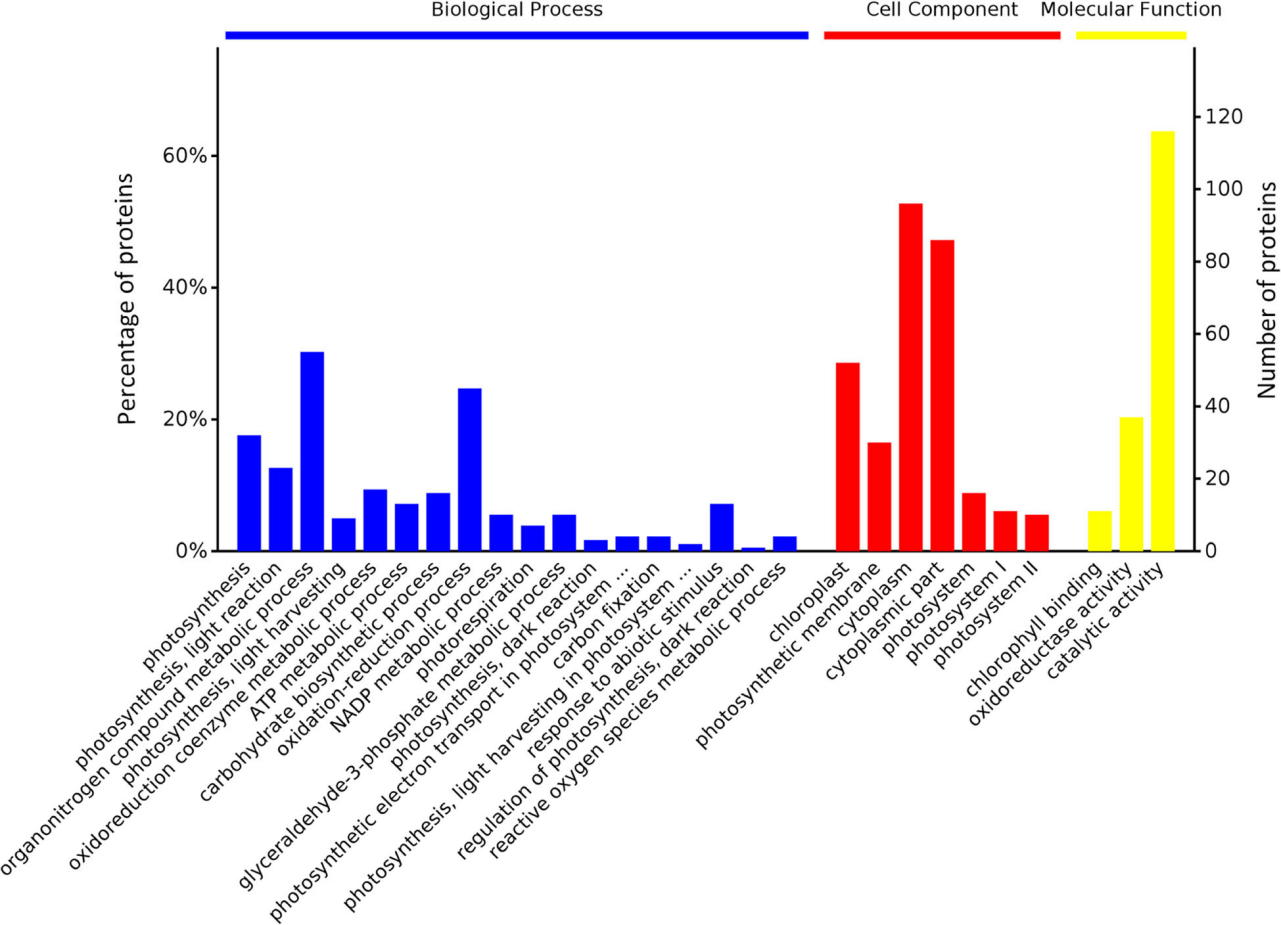
Gene Ontology (GO) enrichment analysis overview between NDT-NST at the tillering stage. The horizontal coordinates of each category are sorted from left to right according to the value of *p*-value. The more significant the items are to the left, the number of DEPs contained in each item and the percentage of them in the total number of DEPs are represented by the vertical coordinates

GO analysis annotated 86 DEPs that were further categorized into three groups: biological process, cellular component, and molecular function between NDT and NST. The biological process categories were photosynthesis (11.94%), photosynthesis, light reaction (8.58%), organonitrogen compound metabolic process (20.52%), photosynthesis, light harvesting (3.36%), oxidoreduction coenzyme metabolic process (6.34%), ATP metabolic process (4.85%), carbohydrate biosynthetic process (5.97%), oxidation-reduction process (16.79%), NADP metabolic process (3.73%), photorespiration (2.61%), glyceraldehyde-3-phosphate metabolic process (3.73%), photosynthesis, dark reaction (1.12%), photosynthetic electron transport in photosystem I (1.49%), carbon fixation (1.49%), photosynthesis, light harvesting in photosystem I (0.75%), regulation of photosynthesis, dark reaction (0.37%), response to abiotic stimulus (4.85%), and reactive oxygen species metabolic process (1.49%). The cellular component categories were cytoplasm (31.89%), cytoplasmic part (28.57%), chloroplast (17.28%), photosynthetic membrane (9.97%), photosystem (5.32%), photosystem I (3.65%), and photosystem II (3.32%). The molecular function categories were chlorophyll binding (6.71%), oxidoreductase activity (22.56%), and catalytic activity (70.73%) (Fig. 7).

**Fig. 8:**
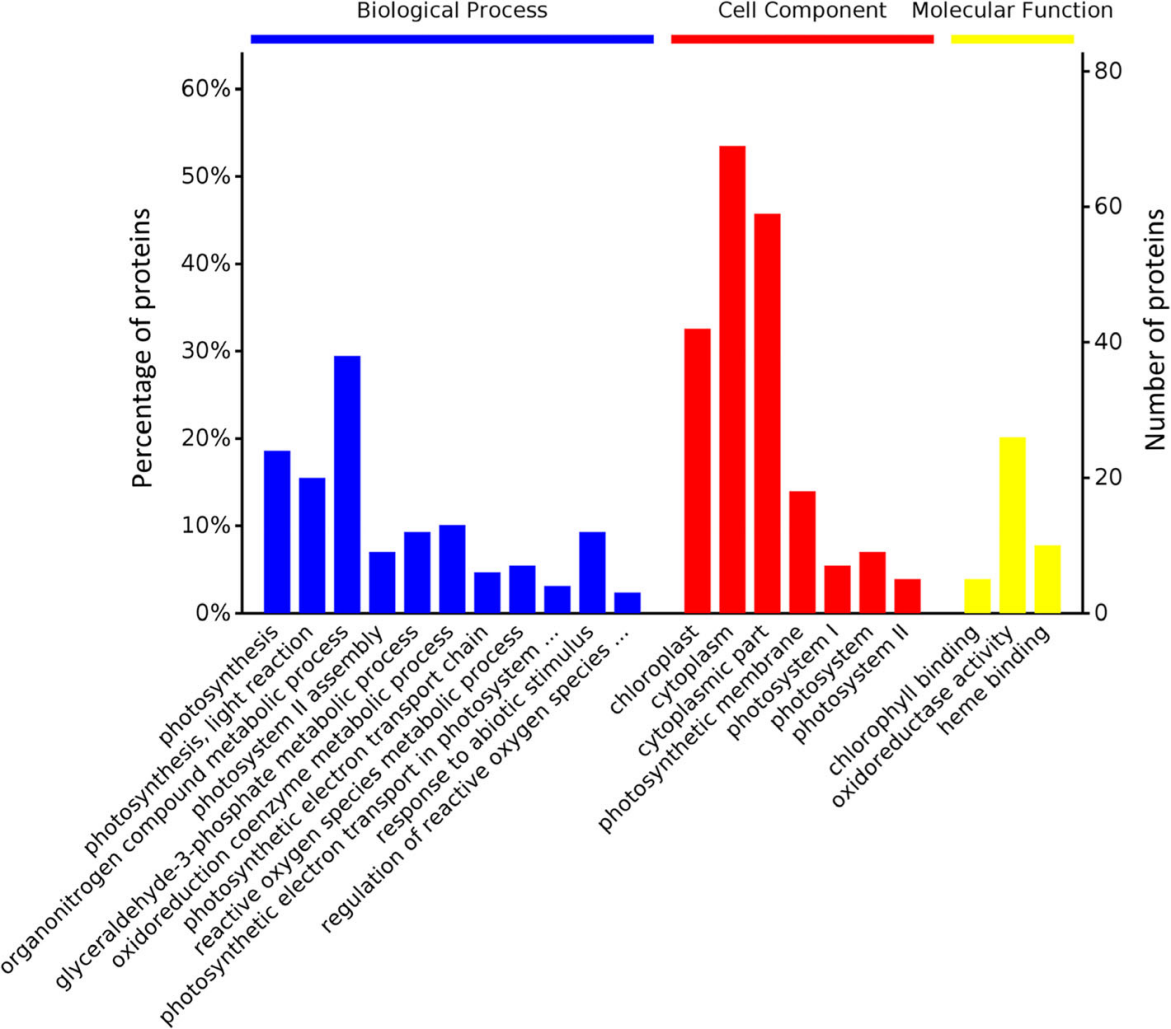
Gene Ontology (GO) enrichment analysis overview between NCP-NSP at the young panicle differentiation stage. The horizontal coordinates of each category are sorted from left to right according to the value of *p*-value. The more significant the items are to the left, the number of DEPs contained in each item and the percentage of them in the total number of DEPs are represented by the vertical coordinates

GO analysis annotated 68 DEPs that were further categorized between NCP and NSP. The biological process categories were photosynthesis (16.22%), photosynthesis, light reaction (13.51%), organonitrogen compound metabolic process (25.68%), photosystem II assembly (6.08%), oxidoreduction coenzyme metabolic process (8.78%), glyceraldehyde-3-phosphate metabolic process (8.11%), photosynthetic electron transport chain (4.05%), ROS metabolic process (4.73%), photosynthetic electron transport in photosystem I (2.7%), response to abiotic stimulus (8.11%), and regulation of reactive oxygen species metabolic process (2.03%). The cellular component categories were cytoplasm (33.01%), cytoplasmic part (28.23%), chloroplast (20.1%), photosynthetic membrane (8.61%), photosystem (4.31%), photosystem I (3.35%), and photosystem II (2.39%). The molecular function categories were chlorophyll binding (12.2%), oxidoreductase activity (63.41%), and heme binding (24.39%) (Fig. 8).

#### Photosynthesis

Photosynthesis includes absorption and transmission of light energy, decomposition of water, charge separation, electron transport and photophosphorylation. In higher plants, the absorption, transfer and transformation of light energy are accomplished by the specific distribution of protein complexes on the thylakoid membrane of chloroplasts and their association with electron carriers [44]. Chlorophyll a-b binding protein is a component of the light harvesting complex (LHC) [45]. Green plants accept solar energy through LHC and assimilate CO_2_, H_2_O and other simple inorganic substances, eventually synthetize complex organic compounds and release oxygen. In this study, chlorophyll a-b binding proteins (I1QEK5, B8AJ38, A2YVI8, Q6H748, A2WUJ5, I1QBM2, A2ZD01, I1PD66, I1PLV0, A2YCB9) were significantly downregulated (*p* < 0.05) under N deficiency (Additional file 7: Table S7). Cytochrome is a class of electron transport proteins with iron porphyrin (or heme) as cogroup, which is involved in the redox reaction in plants. Some types of cellular proteins (heme proteins) play an important role in cell energy transfer. Photosystem I assembly protein Ycf3 (I1QKU5), cytochrome b6-f complex subunit 4 (P0C318), and cytochrome b559 subunit alpha (I1QKV4) are key proteins in photosynthetic electron transport and participate in the synthesis of NADPH [46–49]. NADPH and ATP are generally considered the assimilation capacity of plants. The significant downregulation (*p* < 0.05) of these proteins may result in the reduction of NADPH and ATP synthesis under N deficiency. Rubisco is a key enzyme in photosynthesis that determines the rate of carbon assimilation. Ribulose bisphosphate carboxylase small chain (I1R5Q4) (Additional file 7: Table S7) is an important component of rubisco, which was significantly downregulated (*p* < 0.05) under N deficiency. After N compensation, chlorophyll a-b binding proteins (A2YVI8, Q6H748, I1PD66, I1QEK5, I1QBM2, A2WUJ5) were significantly upregulated (*p* < 0.05) in rice leaves (Additional file 8: Table S8). Cytochrome b6-f complex subunit 4 (P0C318), cytochrome b559 subunit alpha (I1QKV4), NADH-ubiquinone oxidoreductase (Q8W317), and cytochrome b6 (I1PI01) (Additional file 8: Table S8), which are involved in the synthesis of NADPH, were significantly upregulated (*p* < 0.05). Under N deficiency and after N compensation (NCP and NDT), we found the related proteins involved in photosynthesis cytochrome b559 subunit alpha (I1QKV4), light-regulated protein (Q03200), chlorophyll ab binding protein (I1PD66, I1QBM2, Q6H748, I1PLV0), cytochrome b6-f complex subunit (P0C318), photosystem I iron-sulfur center (I1Q6F0), carboxypeptidase (I1PBZ7), nucleoside diphosphate kinase (A6N0M9, I1PYK3), ribulose bisphosphate carboxylase small chain (I1R5Q4), cytochrome b6 (I1PI01) significantly upregulated expression (*p* < 0.05) (Additional file 9: Table S9). The results showed that N deficiency would lead to degradation of pigment proteins in leaves, while N compensation would lead to a large amount of synthesis of pigment proteins in leaves, which was beneficial to the absorption of more solar energy in rice leaves. Thus, the N deficiency and the compensation effect of rice were produced. The photosynthetic proteins function will be further studied under N deficiency and after N compensation.

#### Oxidative stress

Photosystem II activity is attenuated under N deficiency conditions, which results in the accumulation of active oxidizing substances in plant cells in response to adverse environmental stress, and plant cells activate reactive oxygen species (ROS) scavenging systems to protect themselves from harm [50]. ROS scavenging systems in plant cells can be divided into enzymatic and nonenzymatic categories. The former include peroxidase, ascorbate reductase, superoxide dismutase, and catalase and the later include ascorbate and glutathione [51]. NADH dehydrogenase is an enzyme located in mitochondrial inner membrane that catalyzes electron transfer from NADH to coenzyme Q. Our study found that under N deficiency, the key proteins of the rice redox metabolism pathway, including NADH dehydrogenase subunit 5 (I1Q6E7), peroxidase (Q7XIX0, A2Y043), and NADH dehydrogenase subunit 7 (Q8HCQ3) proteins, were significantly upregulated (*p* < 0.05) (Additional file 7: Table S7), suggesting that N deficiency stress indirectly led to injury of plant cells by ROS, which has universality in the plant kingdom. As in other plants, low N stress activates the ROS scavenging system in rice [64]. However, this study also found that the abundance of many other stress-responsive proteins, especially those related to biotic stress, such as pathogenesis-related protein (B8BMF9) (Additional file 7: Table S7), also changed significantly. The results showed that these elements were involved in the processes of N deficiency and biotic stress in plant cells, and many of the elements in these processes intersected. At the same time, we also found that some antioxidant enzymes were significantly upregulated, which provided a solid guarantee for the antioxidant system of rice plants.

#### General metabolism

To analyze the metabolism pathways involved in eliciting a response to N deficiency and N compensation, 86 DEPs were further analyzed by using the KEGG database between NDT and NST. In addition, 68 DEPs were further analyzed using the KEGG database between NCP and NSP. The results showed that most proteins were enriched in photosynthesis, photosynthesis-antenna proteins, metabolic pathways, carbon metabolism, and carbon fixation in photosynthetic organisms between NDT and NST, NCP and NSP, NCP and NDT (Table 4). The only enriched metabolic pathway was starch and sucrose metabolism, glycolysis/gluconeogenesis between NCP and NDT (Table 4). Energy metabolism in plants is the material foundation of the normal growth, development and high yield of crops. Phosphoglycerate kinase (PGK) is a key enzyme of glycolysis and an essential enzyme for each organism to survive, and a lack of the enzyme can cause disorders of metabolism and other functions [52, 53]. The results indicated that the expression of PGK (A0A0P0WP33) was significantly downregulated (*p* < 0.05) under N deficiency (Additional file 7: Table S7), which may be the key position of the limiting glycolysis pathway, resulting in insufficient energy supply under N deficiency at the tillering stage. Glyceraldehyde-3-phosphate dehydrogenase (GAPDH) is a key enzyme in the glycolysis pathway and is closely related to ATP synthesis [54]. Recent studies have shown that GAPDH is not a purely glycolytic enzyme, which is not only involved in energy metabolism but also has many other physiological functions. GAPDH is a multifunctional protein involved in many subcellular activities [55]. In this study, the activity of GAPDH was still significantly downregulated (*p* < 0.05) after N compensation at young panicle differentiation (Additional file 8: Table S8; Additional file 9: Table S9), which may be related to the involvement of GAPDH in other life activities.

**Table 4:** Enriched KEGG pathways associated with DEPs

Pathway Name	Pathway ID	Number of proteins		*p*-value
		Mapping	All	
KEGG pathways between NDT and NST				
Photosynthesis	osa00195	13	70	2.64E-08
Metabolic pathways	osa01100	70	1560	3.07E-08
Photosynthesis – antenna proteins	osa00196	7	15	4.72E-08
Carbon metabolism	osa01200	21	235	7.46E-07
Carbon fixation in photosynthetic organisms	osa00710	9	73	1.28E-04
Glyoxylate and dicarboxylate metabolism	osa00630	8	63	2.50E-04
Pyruvate metabolism	osa00620	8	77	9.92E-04
Oxidative phosphorylation	osa00190	11	137	1.04E-03
Citrate cycle (TCA cycle)	osa00020	6	51	2.29E-03
Glutathione metabolism	osa00480	5	73	4.65E-02
KEGG pathways between NCP and NSP				
Photosynthesis	osa00195	7	70	3.92E-05
Photosynthesis - antenna proteins	osa00196	4	15	3.95E-05
Metabolic pathways	osa01100	32	1560	2.90E-03
Carbon metabolism	osa01200	9	235	4.29E-03
Biosynthesis of amino acids	osa01230	8	207	6.78E-03
Sulfur metabolism	osa00920	3	35	1.19E-02
Porphyrin and chlorophyll metabolism	osa00860	3	37	1.39E-02
Carbon fixation in photosynthetic organisms	osa00710	4	73	1.74E-02
Glycine, serine and threonine metabolism	osa00260	3	57	4.30E-02
KEGG pathways between NCP and NDT				
Photosynthesis	osa00195	1.53E-13	23	70
Metabolic pathways	osa01100	3.63E-07	115	1560
Photosynthesis - antenna proteins	osa00196	5.94E-05	6	15
Carbon fixation in photosynthetic organisms	osa00710	6.93E-05	13	73
Carbon metabolism	osa01200	8.34E-04	24	235
Oxidative phosphorylation	osa00190	4.19E-03	15	137
Biosynthesis of secondary metabolites	osa01110	6.57E-03	58	834
Citrate cycle (TCA cycle)	osa00020	2.68E-01	4	51
Starch and sucrose metabolism	osa00500	4.30E-01	9	158
Glycolysis / Gluconeogenesis	osa00010	7.71E-01	5	123

The pathway ranking in this table is in order from highest to lowest between NDT and NST and between NCP and NSP. The “mapping” number represents the number of annotated DEPs in the pathway, while the “all” number represents the total number of proteins in the pathway. NST: normal N supply at the tillering stage, NDT: N deficiency at the tillering stage, NSP: normal N supply at the young panicle differentiation stage, NCP: N compensation at the young panicle differentiation stage

#### Protein–protein interaction

All annotated functional DEPs were used to analyze protein interactions. This approach revealed that most enzymatic proteins and photosynthesis, metabolic pathways, photosynthesis-antenna proteins, carbon metabolism, and carbon fixation in photosynthetic organism-related proteins interact with NDT and NST (Additional file 10: Figure S1). Most enzymatic proteins and photosynthesis, photosynthesis-antenna proteins, metabolic pathways, carbon metabolism, and biosynthesis of amino acid-related proteins interact with NCP and NSP (Additional file 11: Figure S2). Most enzymatic proteins and photosynthesis, metabolic pathways, photosynthesis - antenna proteins, carbon fixation in photosynthetic organisms, glyoxylate and dicarboxylate metabolism, carbon metabolism-related proteins interact with NCP and NDT (Additional file 12: Figure S3). As a major nutrient in the later growth stage of rice, leaf photosynthesis-assimilated substances play an important role in yield. In this study, the photosynthetic pathway and the photosynthesis-antenna protein pathway were observed to be highly enriched under N deficiency (Additional file 10: Figure S1; Table 4). The pathways were also most enriched after N compensation (Additional file 11: Figure S2; Table 4). This finding suggests that N deficiency and N compensation have important regulatory effects on the photosynthetic capacity of rice leaves. Consistent with our GO analysis findings, the majority of proteins were involved in photosynthesis and metabolic processes (Figs. 6 and 7). We exclusively focused on photosynthesis and metabolic process-related proteins at the proteomic level.

## Conclusion

In this study, we shed new light into the metabolic and protein mechanisms of rice response to N deficiency at the tillering stage and N compensation at the young panicle differentiation stage. The yield per plant exhibited an equivalent compensatory effect. After N compensation, the effective panicle number per plant increased significantly. The net photosynthetic rate, Fv/Fm, SPAD value, and GPT activity were also increased to a certain extent. GO and KEGG enrichment analysis revealed that the DEPs were significantly associated with the photosynthesis pathway, energy metabolism pathway and stress resistance-related proteins. These results are pivotal to future studies that evaluate the impact of N deficiency and the compensation effect on yield formation and provide a new ecological perspective for N utilization in rice.

## Additional files

Additional file 1: **Table S1.** Proteins identified and quantified in experiments 1. (XLSX 222 kb)

Additional file 2: **Table S2.** Information on all the peptides identified in experiment 1. (XLSX 1857 kb)

Additional file 3: **Table S3.** Proteins identified and quantified in experiment 2. (XLSX 243 kb)

Additional file 4: **Table S4.** Information on all the peptides identified in experiment 2. (XLSX 1957 kb)

Additional file 5: **Table S5.** Proteins identified and quantified in experiments 3. (XLSX 248 kb)

Additional file 6: **Table S6.** Information on all the peptides identified in experiment 3. (XLSX 1988 kb)

Additional file 7: **Table S7.** Identification information of N deficiency-responsive DEPs between NDT and NST at the tillering stage. (XLSX 31 kb)

Additional file 8: **Table S8.** Identification information of N compensation-responsive DEPs between NCP and NSP at the young panicle differentiation stage. (XLSX 25 kb)

Additional file 9: **Table S9.** Identification information of N deficiency and N compensation-responsive DEPs between NCP and NDT. (XLSX 62 kb)

Additional file 10: **Figure S1.** DEP protein–protein interaction analysis between NDT and NST at the tillering stage. (DOCX 1128 kb)

Additional file 11: **Figure S2.** DEP protein–protein interaction analysis between NCP and NSP at the young panicle differentiation stage. (DOCX 265 kb)

Additional file 12: **Figure S3.** DEP protein–protein interaction analysis between NCP and NDT. (DOCX 525 kb)

## Data Availability

The mass spectrometry proteomics data have been deposited to the ProteomeXchange Consortium (http://proteomecentral.proteomexchange.org) via the iProX partner repository [56] with the dataset identifier PXD013206.

## Abbreviations

DEPs: Differentially expressed proteins
FC: Fold change
Fv/Fm: Optimal/Maximal quantum yield of photosystem II
GAPDH: Glyceraldehyde-3-phosphate dehydrogenase
GO: Gene ontology
GPT: Glutamic pyruvic transaminase
iTRAQ: Isobaric tags for relative and absolute quantification
KEGG: Kyoto encyclopedia of genes and genomes
LC-MS: Liquid chromatograph-mass spectrometry
N: Nitrogen
NCP: N compensation at the young panicle differentiation stage
NDT: N deficiency at the tillering stage
NSP: Normal N supply at the panicle differentiation stage
NST: Normal N supply at the tillering stage
PCA: Principle component analysis
PGK: Phosphoglycerate kinase
ROS: Reactive oxygen species
SDS: Sodium dodecyl sμLfonate
TEAB: Triethylammonium bicarbonate
