# Supplementary material for: iTRAQ-based quantitative proteomic and physiological analysis of the response to N deficiency and the compensation effect in rice
Source: BMC Genomics. 2019 Aug 28;20:681. doi: 10.1186/s12864-019-6031-4 (PMC6714431; doi:10.1186/s12864-019-6031-4)
Supplement: Supplementary file 11 — Figure S2. DEP protein–protein interaction analysis between NCP and NSP at the young panicle differentiation stage. (DOCX 265 kb) [file 12864_2019_6031_MOESM11_ESM.docx]

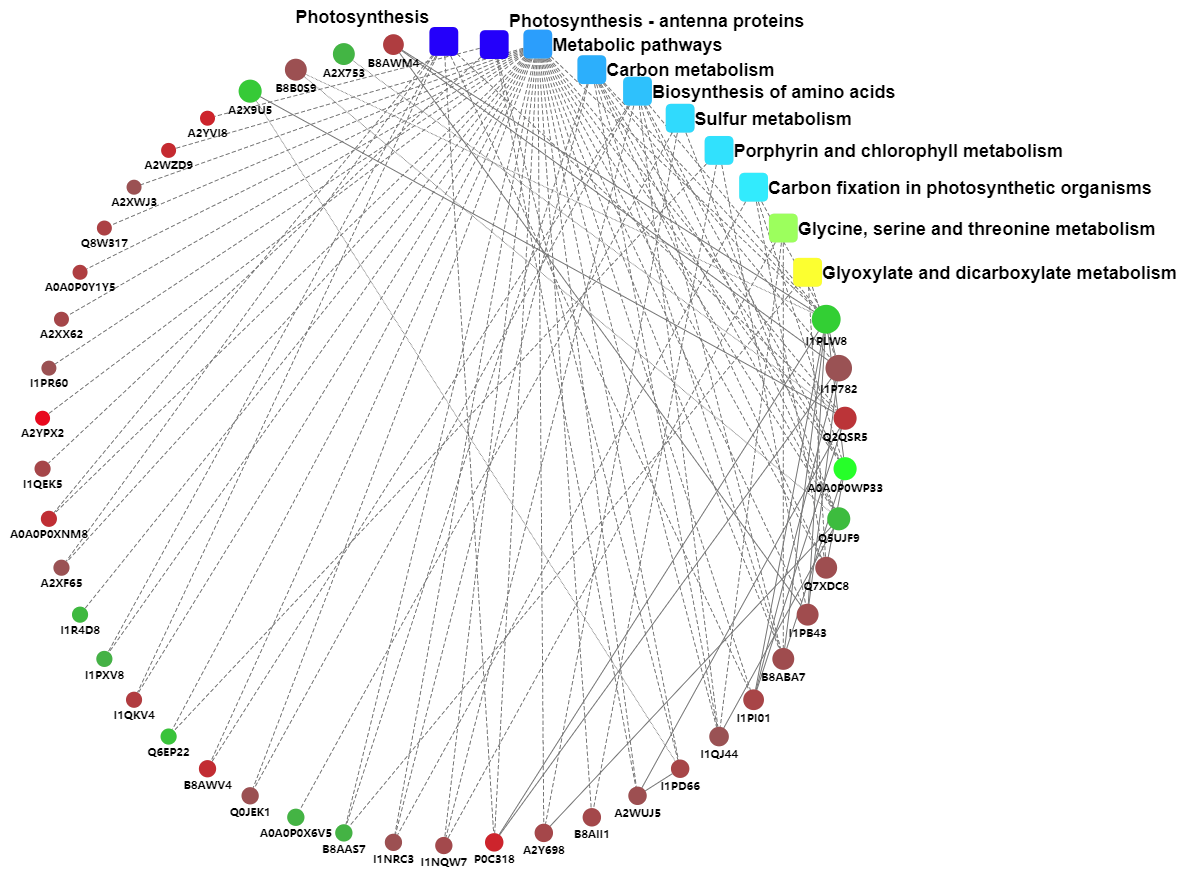


**Fig. S2** DEPs protein–protein interaction analysis between NCP and NSP at the young panicle differentiation stage. Rectangular frames represent different KEGG pathways; blue and yellow represent high and low *p*-value, respectively. Round dots represent proteins, red and green colors indicate increased and decreased relative expressed level of proteins, respectively. NSP: normal N supply at the young panicle differentiation stage, NCP: N compensation at the young panicle differentiation stage.
