# Supplementary material for: iTRAQ-based quantitative proteomic and physiological analysis of the response to N deficiency and the compensation effect in rice
Source: BMC Genomics. 2019 Aug 28;20:681. doi: 10.1186/s12864-019-6031-4 (PMC6714431; doi:10.1186/s12864-019-6031-4)
Supplement: Supplementary file 12 — Figure S3. DEP protein–protein interaction analysis between NCP and NDT. (DOCX 525 kb) [file 12864_2019_6031_MOESM12_ESM.docx]

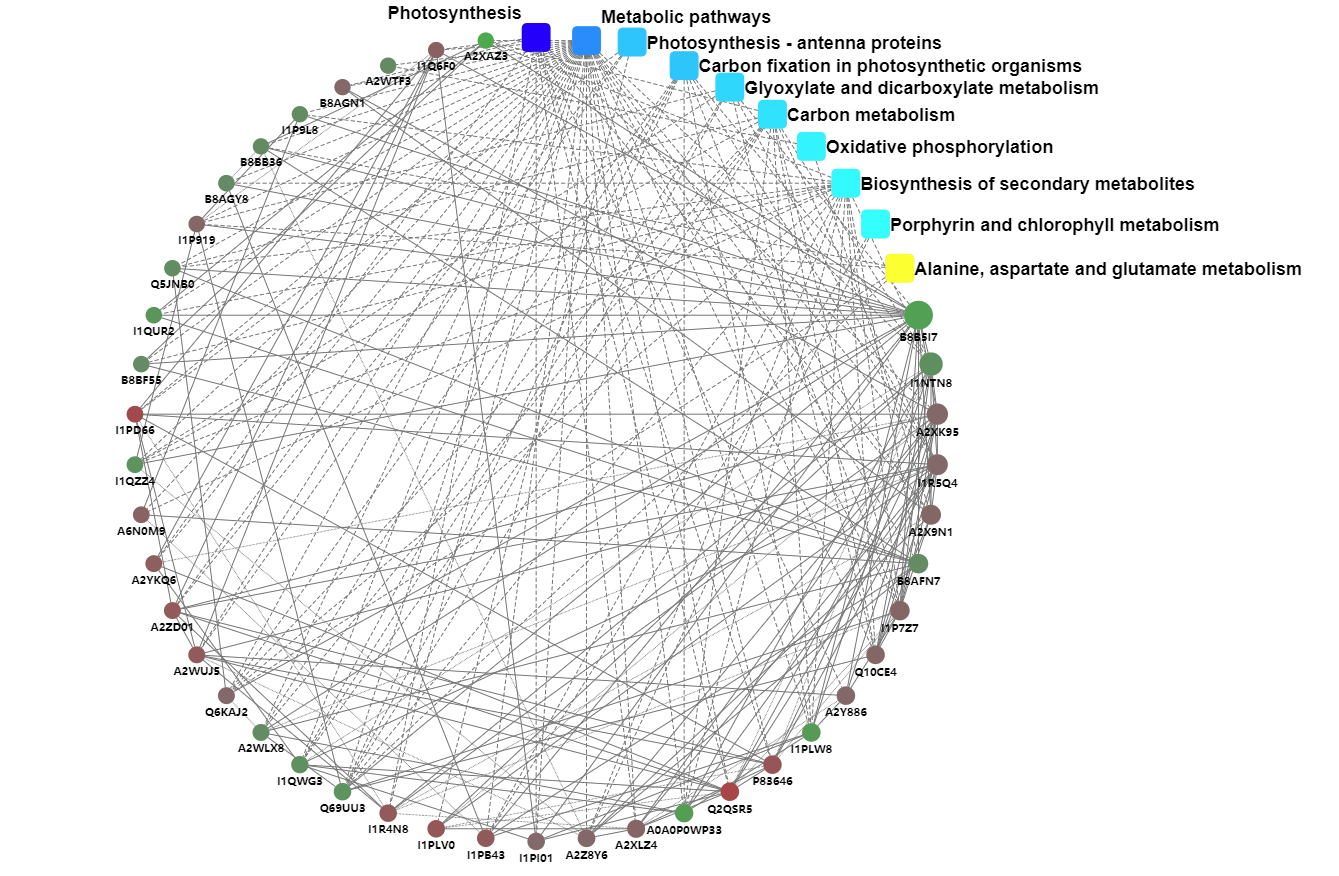


**Fig. S3** DEPs protein–protein interaction analysis between NCP and NDT. Rectangular frames represent different KEGG pathways; blue and yellow represent high and low *p*-value, respectively. Round dots represent proteins, red and green colors indicate increased and decreased relative expressed level of proteins, respectively. NDT: N deficiency at the tillering stage, NCP: N compensation at the young panicle differentiation stage.
